# Whole-Body Electromyostimulation to Fight Osteopenia in Elderly Females: The Randomized Controlled Training and Electrostimulation Trial (TEST-III)

**DOI:** 10.1155/2015/643520

**Published:** 2015-02-15

**Authors:** Simon von Stengel, Michael Bebenek, Klaus Engelke, Wolfgang Kemmler

**Affiliations:** Institute of Medical Physics, University of Erlangen-Nürnberg, 91052 Erlangen, Germany

## Abstract

Whole-body electromyostimulation (WB-EMS) has been shown to be effective in increasing muscle strength and mass in elderly women. Because of the interaction of muscles and bones, these adaptions might be related to changes in bone parameters. 76 community-living osteopenic women 70 years and older were randomly assigned to either a WB-EMS group (*n* = 38) or a control group (CG: *n* = 38). The WB-EMS group performed 3 sessions every 14 days for one year while the CG performed gymnastics containing identical exercises without EMS. Primary study endpoints were bone mineral density (BMD) at lumbar spine (LS) and total hip (thip) as assessed by DXA. After 54 weeks of intervention, borderline nonsignificant intergroup differences were determined for LS-BMD (WB-EMS: 0.6 ± 2.5% versus CG −0.7 ± 2.5%, *P* = .051) but not for thip-BMD (WB-EMS: −1.1 ± 1.9% versus CG: −0.8 ± 2.3%, *P* = .771). With respect to secondary endpoints, there was a gain in lean body mass (LBM) of 1.5% (*P* = .006) and an increase in grip strength of 8.4% (*P* = .000) in the WB-EMS group compared to CG. WB-EMS effects on bone are less pronounced than previously reported effects on muscle mass. However, for subjects unable or unwilling to perform intense exercise programs, WB-EMS may be an option for maintaining BMD at the LS.

## 1. Introduction

Exercise maintains bone mass. But do people maintain exercise? [1]. Indeed, the majority of elderly subjects in Germany [2] or the US [3] fall far short of the exercise doses recommended for consistently impacting Bone Mineral Density [4–6]. A novel training technology, called whole-body electromyostimulation (WB-EMS), may increase effects of moderate exercise on the musculoskeletal system and thus might be a time-saving and feasible option for subjects unable or unwilling to perform strenuous conventional exercise. Unlike local EMS application, WB-EMS technology simultaneously stimulates up to 14–18 regions or 8–12 muscle groups with up to 2.800 cm^2^ electrode area. Although EMS-technology primarily focuses on muscle by activating muscle contraction and directly stimulating muscle protein synthesis rate [7], there is some evidence that this mode of muscle stimulation also impacts bone [8].

However, human trials determined the effect of EMS-application on bone only in the case of disuse-induced bone loss in spinal cord injury (SCI) patients. In these studies EMS was applied either locally only on plantarflexors or as functional electrostimulation (FES) activating isolated thigh muscles to induce leg cycling movements on ergometry or knee extention movements on strength machines. One meta-analysis [9] and two reviews [10, 11] analyzed and discussed the effect of EMS and FES on BMD in SCI and found positive results. Basic mechanisms of bone adaptions to disuse after SCI and following EMS training are reviewed by Dudley-Javoroski and Shields [12]. However, conclusions drawn from studies determining the effect of EMS on bone in the specific situation of disuse in paralyzed patients are not valid for the normal population and, furthermore, the transferability of results of local EMS studies on the WB-EMS technology is rather questionable.

In the present study we evaluated the impact of WB-EMS on BMD in sedentary, lean, and osteopenic elderly females, a cohort at high risk for fractures. The main hypothesis of this trial was that WB-EMS training significantly increases BMD at the lumbar spine compared with a control group. Our secondary hypothesis was that WB-EMS training significantly impacts BMD at the proximal femur compared with the control group. Secondary endpoints concerning body composition and strength are published elsewhere [13].

## 2. Materials and Methods

The Training and Electrostimulation Trial (TEST) project is a series of studies that determine the effect of WB-EMS on parameters related to risk factors and diseases of elderly subjects. In the present study, TEST-III is a randomized, controlled trial (RCT) that evaluated the effect of whole-body electromyostimulation (WB-EMS) on osteoporosis in lean, sedentary elderly females with osteopenia. To determine the isolated effect of WB-EMS we implemented an active control group which performed identical movements as carried out during the WB-EMS session. The study protocol was approved by the Ethical Committee of the Friedrich-Alexander University Erlangen-Nuremberg (FAU), Germany (Ethik Antrag 4184), and the German Radiation Safety Agency (Z 5-22462/2-2010-027). Written informed consent was obtained from all subjects prior to the study entry. TEST-III was conducted from November 2010 through July 2012 at the Institute of Medical Physics, FAU, Germany, and is fully registered under www.clinicaltrials.gov (NCT01296776).

### 2.1. Participants

**Figure 1:**
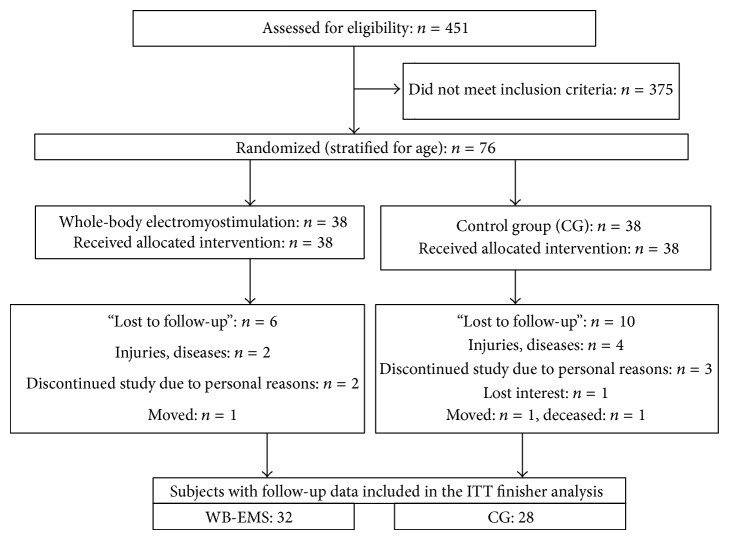
Flow chart of the “TEST-III” study (CONSORT scheme).

All female subjects (9256 women) 70 years and older, living in the area of Erlangen, Germany, were contacted by personal letters. Though eligibility criteria for the trial (Figure 1) already had been listed in the letter, 272 subjects of the 451 women who responded had to be excluded after phone interviews, because they (a) did not meet our criteria for “leanness” (body weight (kg) > (body height (cm) − 100)), (b) had exercised more than 1 hour per week during the last 10 years, (c) reported contraindications (i.e., total endoprosthesis, abdomen/groin hernia, epilepsy, and cardiac arrhythmia) for WB-EMS intervention, and (d) reported diseases or medication affecting our primary endpoints.

179 women were invited to our lab to determine body composition and bone status. After measuring body weight and height with calibrated devices and determining BMD at the lumbar spine and proximal femur, 103 women had to be excluded because they did not meet our inclusion criterions of “leanness” or osteopenia (BMD < 1 SD T-Score) (Figure 1).

The 76 remaining subjects were assigned into two study groups using computerized block randomization stratified for age (block size: *n* = 2) by an external statistician to ensure allocation concealment.  Table 1 lists baseline characteristics of the WB-EMS and the control group. No corresponding intergroup differences were observed.

### 2.2. Interventions

WB-EMS group performed 54 weeks of consecutive WB-EMS intervention, while in parallel the control group (CG) carried out an intermitted not strenuous gymnastics program. The underlying rationale for this procedure was to validate the isolated effect of WB-EMS versus a motivated and “blinded” control group that performed gymnastics containing identical low intensity/low amplitude movements as the WB-EMS group.

Both interventions were performed at the Institute of Medical Physics (IMP), which could be easily reached by the subjects. All the sessions were supervised by certified trainers who recorded the attendance of the participants.

In order to check for parameters that may impact our study endpoints, lifestyle parameters (i.e., dietary intake, physical activity, etc.) were inquired by questionnaires at baseline and follow-up [16, 17]. After analyzing standardized dietary intake protocols over 4 days (Prodi-4.5, Wissenschaftlicher Verlag, Freiburg, Germany), both groups were provided with a maximum of 1,200 mg/d calcium and 800 IU/d of cholecalciferol (Rottapharm/Madaus, Cologne, Germany).

Apart from these interventions, subjects were asked to maintain their habitual life style.

### 2.3. WB-EMS Intervention

The WB-EMS exercise protocol is described in more detail in another publication [18]. The WB-EMS group performed supervised WB-EMS training (18-19 min/session; bipolar current; frequency: 85 Hz; pulse-breadth: 350 *μ*sec) 3 times in 2 weeks (each Monday or Tuesday and every second Thursday or Friday) for 54 weeks using the WB-EMS technology of miha bodytec (Augsburg, Germany). Eight muscle groups were simultaneously activated by electrostimulation: upper legs, upper arms, bottom, abdomen, chest, lower back, upper back, and latissimus dorsi. WB-EMS sessions consisted of easy, not strenuous dynamic exercises performed in a standing position, with 6 s dynamic movements under EMS intermitted by 4 s of static rest without current. 10–14 exercises (e.g., dead lift with elbow-extension or arm-flexion; squat with trunk flexion; squat with lat pulleys or military press; squat with crunch and butterfly or reverse fly; squat with vertical chest press or vertical rowing) were structured in 1-2 sets of 8 repetitions. Total time under load (current) averaged ≈11 min/session during which ≈110 stimulation intervals à 6 sec were completed. Motion amplitude as well as corresponding intensity generated by the movements was set low (i.e., squat: leg-flexion: <35°) to prevent effects from the exercise per se.

**Figure 2:**
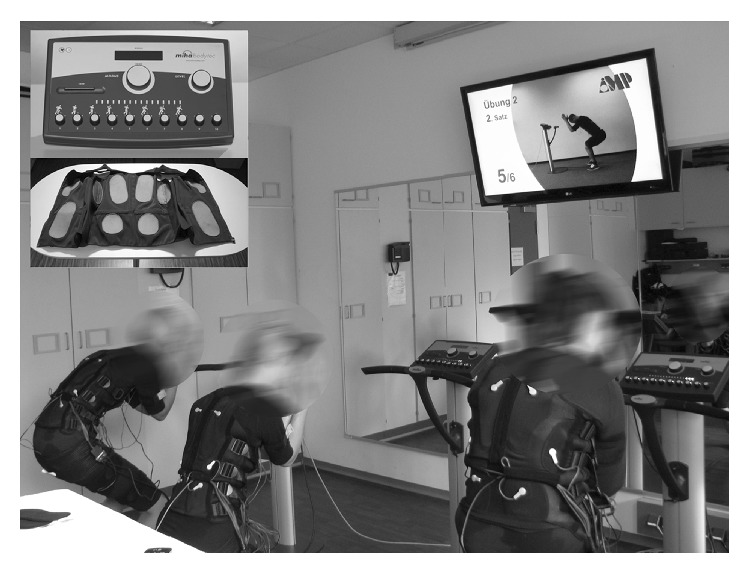
Whole-body electromyostimulation equipment.

In a WB-EMS session 3 subjects underwent supervised and video-guided exercise (at three WB-EMS devices) at the same time. Strong emphasis was placed on adequate (current) intensity. Current intensity was individually adapted for each region during the first sessions to a Rate of Perceived Exertion (RPE) of 14–16 of 20 (“somewhat hard” to “hard”). Intensities were saved on chip cards that allowed fast and reliable setting of the devices in the subsequent sessions. If necessary, current intensity was increased in the following sessions to maintain the predefined rate of perceived exertion.  Figure 2 illustrates the WB-EMS equipment and training.

### 2.4. Control Group (CG)

Subjects of the CG performed two 10-week blocks of easy, not strenuous gymnastics with 1 session (60 min)/week with 10 weeks of rest between the blocks. After 5 min of walking variations, subjects performed the identical low intensity/low amplitude dynamic exercises as during the EMS sessions. After this, flexibility, general coordination, and balance exercises were carried out for 20 min. The session finished with 15 min of relaxation and communication. The aim of the CG protocol was to ensure a “blinding” by carrying out a multifaceted, attractive exercise and relaxation program. The program was designed not to impact the study endpoints bone and muscle.

### 2.5. Endpoints

Primary and secondary endpoints were determined at baseline and after 54 weeks of intervention.

*Primary Outcome Measures*

- Bone Mineral Density at the lumbar spine (LS),
- Bone Mineral Density at the proximal femur (total hip region of interest (thip-ROI)).

*Secondary Outcome Measures*

- Total lean body mass (LBM),
- grip strength.

### 2.6. Testing Procedures

All assessments and analysis were carried out in a blinded mode. Research assistants were not informed about the status of the participants (WB-EMS or CG) and were not allowed to ask either.

### 2.7. Anthropometry

Body weight was measured to the nearest 0.1 kg on a digital scale (InBody 230, Seoul, Korea). Height was determined barefoot to the nearest 0.1 cm with a stadiometer (Holtain, Crymych Dyfed, Great Britain). Body composition was assessed with Dual Energy X-Ray Absorptiometry (DXA, QDR 4500a, Discovery-upgrade; Hologic Inc., Bedford, USA) using standard protocols. Grip strength of the dominant arm was assessed with a Jamar dynamometer (Jamar, Bolington, IL).

Bone Mineral Density (BMD) was also determined by DXA at the lumbar spine (L1–L4, a.p.) and the proximal femur (total hip ROI) at baseline and after 1 year using standard protocols specified by the manufacturer. Long-term reliability was 0.4% (coefficient of variation) as determined by 177 lumbar spine phantom scans conducted during the study period.

### 2.8. Statistical Analyses

The sample size calculation was based on the study endpoint “Bone Mineral Density changes at the lumbar spine.” In order to detect a relevant between-group difference of 2.0% (Standard Deviation (SD): 2.8%) 31 subjects/group were required (5% error probability, 80% statistical power). Analyses were performed on an intention-to-treat basis for all the participants who completed the baseline and at least one follow-up measurement (finisher analysis). The treatment effect was defined as between-group differences in changes from baseline to 12 months. In order to obtain normally distributed data all endpoints (BMD, LBM) were log-transformed. Analysis of covariance with baseline value, age, height, and fat and muscle mass as covariates were carried for statistical comparison of the two study groups. Statistical significance was accepted at *P* < 0.05 (2-tailed). Effect sizes were calculated using Cohen's d [19]. We used SPSS 19.0 (SPSS Inc, Chicago, IL) for all the statistical procedures.

## 3. Results

16 (WB-EMS, *n* = 6; CG, *n* = 10) of 76 subjects were unable or unwilling to visit the one-year control assessment and were thus lost to follow-up (Figure 1). Altogether 6 women suffered fractures, surgery, and/or serious diseases (cancer, CHD). One subject of the CG lost interest, and five subjects listed personal reasons for their withdrawal, two of them related to the CG protocol, one related to the WB-EMS protocol. One participant of the CG died during the interventional period and one woman of each group moved. Thus 28 subjects of the CG (74%) and 32 subjects of the WB-EMS group (84%) were included in the analysis. No serious training-related adverse effects were observed during the sessions.

Summing up, as total attendance in the WB-EMS group, 61 out of 78 sessions (79 ± 18%) were conducted. The corresponding attendance rate in the CG was slightly lower (74 ± 18%; 14.9 of 20 sessions). Although the total number of sessions was much lower in the CG, the difference in total exercise volume was smaller with an average of ≈24 min/week in the WB-EMS group and ≈18 min in the CG.

No intergroup differences were recorded for anthropometric and clinical parameters at baseline (Table 1). With respect to confounding parameters, no significant changes between baseline and follow-up were observed for lifestyle parameters (physical activity, e.g.) and/or medication as recorded by questionnaires or dietary intake as assessed by 4-day dietary protocol and analyzed using Prodi-4.5/03 Expert software (Wissenschaftlicher Verlag, Freiburg, Germany).

### 3.1. Primary and Secondary Outcome Measures

BMD at the LS increased in the WB-EMS groups (0.6 ± 2.5%) and decreased in the CG (−0.6 ± 2.4%). At the total hip ROI BMD decreased in both groups (WB-EMS: −0.9 ± 1.9% versus CG: −1.0 ± 2.3%). Similar negative changes were observed for the proximal femur subregions (femoral neck, trochanter). Differences between WB-EMS and CG-group were borderline nonsignificant for LS-BMD (*P* = 0.051, ES: *d*′ = 0.49) and not significant for BMD of the total hip ROI (*P* = 0.771, ES: *d*′ = 0.04).

Total LBM as assessed by total body DXA scans increased in the WB-EMS group (0.7 ± 1.6%) and decreased in the CG (−0.8 ± 2.5%). Corresponding differences between WB-EMS and CG-group were significant (*P* = 0.006, ES: *d*′ = 0.71).

The changes in grip strength were 10.5 ± 12.6% in the WB-EMS group and 2.2 ± 8.19% in the CG (*P* = 0.000, ES: *d*′ = 0.71).  Table 2 lists the changes of BMD and strength parameters in both groups.

## 4. Discussion

This is the first trial that determined the effect of (WB-) EMS on BMD at lumbar spine or/and proximal femur in elderly females with osteopenia. We found a borderline significant effect (*P* = 0.051) for the lumbar spine BMD, but not for the femoral neck site. In view of the high effects of WB-EMS on muscle mass and strength in previous studies [20, 21] and the close interaction of muscle and bone [8], we had expected stronger effects on bone. In the present study the effect of WB-EMS on LBM was significant but rather moderate, showing a 1.5% net gain. However, maximum isometric leg and trunk extension strength significantly increased by more than 10%, as published elsewhere [13].

All the other studies examining the effect of EMS in BMD exclusively determined (functional) electromyostimulation (FEMS) under disuse conditions (e.g., SCI) [22–32]. In the recent meta-analysis by Chang et al. [9] the authors found a significant increase in BMD after 3, 6, and 12 months of FES leg cycling or FES knee extension exercises in SCI patients. A longer period of exercise and a higher training frequency were associated with higher effectiveness. Dolbow et al. [10] reviewed 10 studies determining the effect of FES leg cycling on BMD, taking into account the time after injury. Two of two studies report effects of FES therapy in the first 2 months after injury; only one of three studies showed effects at an average of 3–6 years after injury and 4 out of five 9–13 years after injury. In line with the results of Dolbow et al., Biering-Sørensen et al. [11] concluded in a systematic review including 19 EMS studies that there may be some effects of electrical stimulation especially in the early phase. This review includes studies with EMS of the lower limb (4 studies), leg cycling FES (7 studies), knee extention FES (5 studies), FES during treadmill gait (1 study), or a combination of leg cycle and knee extension FES. Consistent with the results of Chang et al. improvement is seen in a longer period of training or with higher training frequency.

Although the studies included in the meta-analysis and reviews differ widely with respect to subjects or measurements, the large majority of trials which ensured sufficient time for bone adaption (6–12 months) reported positive BMD changes at skeletal sites stimulated and loaded by EMS or FES cycling or FES leg-extension exercises. However, it can be assumed that in paralyzed subjects the strain threshold is low due to inactivity and it remains unclear if the positive BMD changes were caused more by EMS induced muscle contraction producing joint reaction forces or just by resulting external reaction forces leading to axial loading of bones during exercises like leg-extension or FEMS cycling. And again, the relevance of the results of these studies for older people without serve functional limitations is rather questionable.

A comparison of the effect of WB-EMS with “gold-standard exercise protocols” might be helpful for estimating the relevance of WB-EMS programs for fighting osteoporosis. Reviewing the literature for exercise-induced BMD changes in subjects 60 years and older as assessed by DXA (review [33, 34]) shows that our WB-EMS effects were lower compared with conventional exercise effects in particular when specific resistance exercise was applied. Comparing the results of the present WB-EMS results with data of our recent 18-month SEFIP exercise trial [35] that used identical measurements and included comparable subjects with respect to age (69 ± 4 yrs) with higher BMI (26 ± 4 kg/m^2^) revealed more favorable data for the SEFIP cohort (net BMD difference EG versus CG at LS and thip ≈1.5–2.1%; both *P* = 0.001) that had carried out a combined resistance/aerobic/balance protocol.

Little is known about the optimum EMS protocol for impacting bone, and the low effect on Bone Mineral Density in the present study might be due to a suboptimal setting of current parameters. Although the most favorable composition of EMS parameters for triggering bone adaptation still has to be established, a recent study that directly compared different frequencies of EMS in an animal disuse model [36] determined the most favorable effect on bone parameters (e.g., volume fraction, connectivity, and trabecular number/thickness) especially at 20 and 50 Hz. With respect to stimulation intensity Dudley-Javoroski and Shields [12] suggested supramaximal amperage (200 mA) to elicit very strong contractions. Because each pair of electrodes was regulated separately with different intensities and due to differences in electrode size, we were unable to control and describe the stimulation intensity (mA) in the present study. In EMS studies in SCI patients, where isolated muscles were stimulated, external forces were measured and put into relation with body weight to estimate intensity [31]. Because of the simultaneous activation of agonists and antagonists and resulting co-contraction, this method cannot be used in WB-EMS. We applied EMS for 3 × 20 min in 2 weeks using a bipolar current at 85 Hz at a pulse width of 350 *μ*sec whereas 6 sec of stimulation was intermitted by 4 sec of rest. The highly significant effect of this protocol on muscle mass and strength and its acceptance [20, 21, 37] supported the application of this EMS protocol.

Considering that enthusiasm for conventional exercise is rather low in the cohort of elderly women, one key factor for the relative high compliance with EMS training [33] may have been the low total volume of the program (in total 30 min/week). Furthermore, WB-EMS programs are applied under rather individualized conditions and it may be also the exclusiveness of the exercise program that resulted in a high compliance.

Some limitations may decrease the evidence of the present study. (a) We evaluated the effect of EMS in the cohort of lean elderly females with osteopenia and the assignability of our results to other cohorts is questionable. (b) Although DXA is still the “gold standard” technology for assessing BMD, QCT technique may be the more appropriate method for assessing BMD at lumbar spine in the cohort of subjects 70 years and older due to degenerative changes of the spine. (c) No X-ray examinations have been performed to detect vertebral body fractures. However, we presume that BMD increases in WB-EMS group were not the result of “training induced” compression fractures. In the analyzed LS-scans no decrease of area of the vertebra as sign of vertebral deformity was observed. Further, during WB-EMS the mechanical loading of vertebrae due to muscular tension is rather moderate compared to classic high impact training contents. (d) A semiactive control group that performed a comparable exercise volume and identical movements was implemented to ensure blinding and to determine the effect of WB-EMS per se. For reasons of attractiveness and compliance the CG did not carry out only identical movements but also performed exercises for mobility, coordination, and relaxation; and the training schedule differed. However, the movements of the WB-EMS program and all the contents of the CG sessions were not strenuous and designed not to impact our endpoint. Thus, in our opinion, the influences of the differences in exercises contents and schedule on the validity of the studies are rather low. (e) Due to a lower number of subjects included (76 instead of 80) and a slightly higher drop-out rate than expected we failed to realize our estimated sample size of 31 subjects/group. (g) It is not possible to objectify intensity of muscle contraction during WB-EMS and it is not clear if subjects realized the requested high intensity.

## 5. Conclusions

In summary, we found a borderline nonsignificant effect of WB-EMS on Bone Mineral Density at the lumbar spine and no effect at the hip. However, taking into account the high impact of this technology on muscle mass and strength, WB-EMS may be an option for musculoskeletal prevention/rehabilitation at least for (elderly) subjects unable or unwilling to exercise conventionally. Nevertheless, due to the higher impact of mixed exercise programs on BMD and their comprehensive effect on multiple risk factors and diseases of advanced age [38], classic exercise should be favored for elderly subjects.

## Figures and Tables

**Table 1:** Baseline characteristics of the WB-EMS and control group. WB-EMS: whole-body electromyostimulation. ^1^As assessed by detailed questionnaires (14, 15). ^2^As assessed by 4-day protocol and analyzed using Prodi-4.5/03 Expert software (Wissenschaftlicher Verlag, Freiburg, Germany). ^3^As assessed by Jamar dynamometer (dominant hand) according to Mathiowetz et al. [14]. ^4^Test according to Fritz und Lusardi [15]. ^5^Prevalence of two and more diseases.

Variable	WB-EMS (*n* = 38)	CG (*n* = 38)
Age [years]^1^	74.7 ± 3.7	74.7 ± 4.4
Body weight [kg]	57.9 ± 6.8	58.8 ± 5.7
Body length [cm]	161.6 ± 5.6	162.9 ± 5.1
Year postmenopausal [years]	24.3 ± 4.2	25.2 ± 4.7
Total body fat DXA [%]	31.6 ± 4.6	32.1 ± 3.7
Appendicular skeletal muscle mass [kg]	15.8 ± 2.1	15.9 ± 1.7
Energy uptake [MJ/d]^2^	6.63 ± 1.81	6.74 ± 1.67
Calcium uptake [mg/d]^2^	986 ± 276	966 ± 266
Vitamin D uptake [IU/d]^2^	244 ± 167	262 ± 211
Exercise volume [min/week]^1^	34.1 ± 21.6	31.3 ± 19.3
Grip strength^3^ [kg]	23.7 ± 4.0	23.5 ± 4.1
Walking speed^4^ [km/h]	5.1 ± 1.4	5.3 ± 1.6
Multimorbidity^5^ [*n*]	22 (58%)	25 (66%)

**Table 2:** Baseline and follow-up data of the WB-EMS and CG group for BMD lumbar spine (LS), total hip, lean body mass (LBM) and grip strength, absolute treatment effects between training and control, and *P* value (covariance analysis, baseline value, age, height, lean body mass, and fat mass).

	WB-EMS (*n* = 32)		CG (*n* = 28)		Treatment effect	
Group	Baseline	12 months	Baseline	12 months	Mean (95% CI)	*P* value
	Mean (SD)		Mean (SD)		
BMD LS [mg/cm^2^]	882 ± 178	886 ± 173	835 ± 103	830 ± 105	10.4 (−21.3 to 0.5)	0.051
BMD hip [mg/cm^2^]	763 ± 81	756 ± 85	754 ± 95	746 ± 0.097	1.2 (−9.0 to 6.61)	0.771
LBM (Kg)	35.15 ± 4.43	35.42 ± 4.40	35.42 ± 3.52	35.12 ± 3.6	0.57 (0.16 to 0.98)	0.006
Grip strength (Kg)	23.9 ± 4.0	26.41 ± 3.6	23.1 3.9	23.6 ± 4.5	2.07 (0.88 to 3.26)	0.000
